# The Role of STAT3 Signaling Pathway Activation in Subconjunctival Scar Formation after Glaucoma Filtration Surgery

**DOI:** 10.3390/ijms241512210

**Published:** 2023-07-30

**Authors:** Yanxia Li, Jing Zhao, Yuan Yin, Chenchen Zhang, Zhaoying Zhang, Yajuan Zheng

**Affiliations:** Department of Ophthalmology, The Second Hospital of Jilin University, Jilin University, Changchun 130041, China; yanxia20@mails.jlu.edu.cn (Y.L.); lhbswqw@jlu.edu.cn (J.Z.); yinyuan215@jlu.edu.cn (Y.Y.); cczhang21@mails.jlu.edu.cn (C.Z.); zhaoyingz22@mails.jlu.edu.cn (Z.Z.)

**Keywords:** signal transducer and activator of transcription 3 (STAT3), glaucoma filtration surgery, fibrosis, suppressor of cytokine signaling 3 (SOCS3)

## Abstract

Scar formation resulting from overly active wound healing is a critical factor in the success rate of glaucoma filtration surgery (GFS). IL-6 and TGF-β have been implicated in the pathogenesis of fibrogenesis. In addition, the signal transducer and activator of transcription 3 (STAT3) can be activated by numerous cytokines and growth factors, including IL-6 and TGF-β1. Thus, STAT3 activation may integrate common profibrotic pathways to promote fibrosis. In this study, an increase in p-STAT3 was observed in activated HTFs. Inhibiting STAT3 in cultured HTFs by pharmacological inactivation reversed the fibrotic responses, such as fibroblast migration, the differentiation of resting fibroblasts into myofibroblasts and the deposition of ECM, mediated by IL-6 and TGF-β1. Moreover, the expression of suppressor of cytokine signaling 3 (SOCS3) was decreased in HTFs cultured with IL-6 and TGF-β1, and SOCS3 overexpression rescued ECM deposition, α-SMA expression and migration in IL-6- and TGF-β1-stimulated HTFs by inactivating STAT3. Finally, S3I-201 treatment inhibited profibrotic gene expression and subconjunctival fibrosis in a rat model of GFS. In conclusion, our data suggests that STAT3 plays a central role in fibrosis induced by different profibrotic pathways and that STAT3 is a potential target for antifibrotic therapies following GFS.

## 1. Introduction

Glaucoma is a chronic progressive optic neuropathy and a major cause of blindness worldwide. It is anticipated that 111.8 million patients will suffer from glaucoma by 2040 [1]. An increase in intraocular pressure (IOP) is a major risk factor for glaucoma and permanent loss of vision [2]. Currently, lowering IOP through medical and surgical methods remains the only evidence-based treatment available [3]. Glaucoma filtration surgery (GFS), which is a surgical procedure that involves the guidance of aqueous humor into the space between the sclera and conjunctiva, is widely used to lower IOP. However, fibrotic responses such as aberrant fibroblast proliferation, myofibroblast differentiation, and excessive deposition of extracellular matrix (ECM) induced by GFS-induced injury in the subconjunctival area could result in surgical failure [4]. Although antimetabolic agents, such as mitomycin C (MMC), have been commonly used to inhibit scar formation at the filtering site, some patients continue to have poor prognoses after GFS [5]. Additionally, current antifibrotic therapies also have large side-effect profiles, including endophthalmitis, cornea toxicity, blebitis and wound leakage [6,7,8]. Thus, better antifibrotic therapies that can improve the efficacy and safety of GFS are needed to avoid blindness due to glaucoma.

Transforming growth factor β (TGF-β) plays a central role in modulating fibrosis by stimulating fibroblasts and the differentiation of myofibroblasts. Additionally, interleukin-6 (IL-6), which is a multifunctional cytokine involved in inflammatory responses, was shown to promote fibrosis in previous studies [9,10,11]. IL-6 signal transduction involves the classic signaling pathway and trans-signaling pathway, which are mediated by membrane-bound IL-6R (mIL-6R) and soluble IL-6R (sIL-6R), respectively [12,13,14]. Although mIL-6R is expressed only on specific cell types, IL-6 can transduce its signal to nearly all cells through sIL-6R. In response to IL-6 stimulation, signal transducer and activator of transcription 3 (STAT3) proteins are recruited and phosphorylated, and then the homodimerized STAT3 translocates to the nucleus, where it triggers the transcription of various target genes, including cellular differentiation, apoptosis, angiogenic factors and cytokines [15,16]. Interestingly, there is substantial evidence that TGF-β can also activate STAT3 [17,18], suggesting that STAT3 activation may integrate common profibrotic pathways to promote fibrosis.

As a result, there is considerable interest in determining whether STAT3 inhibition alleviates subconjunctival fibrosis following GFS. The purpose of this study was to examine the effect of inhibiting STAT3 on the development of subconjunctival fibrosis in two complementary fibrosis models of human tenon fibroblasts (HTFs): one involving inflammation-dependent pathways (induced by IL-6) and the other involving fibroblast activation devoid of inflammation (induced by TGF-β1). S3I-201, a selective small molecule inhibitor of STAT3 [19], was used to test the efficacy of STAT3 inhibition on the prevention of GFS fibrosis in vivo and in vitro. Our findings provide the first evidence that pharmacological inhibition of STAT3 by S3I-201 prevents scar formation following GFS.

## 2. Results

### 2.1. Phosphorylated STAT3 Is Increased in Activated Fibroblasts Induced by IL-6 and TGF-β1

**Figure 1:**
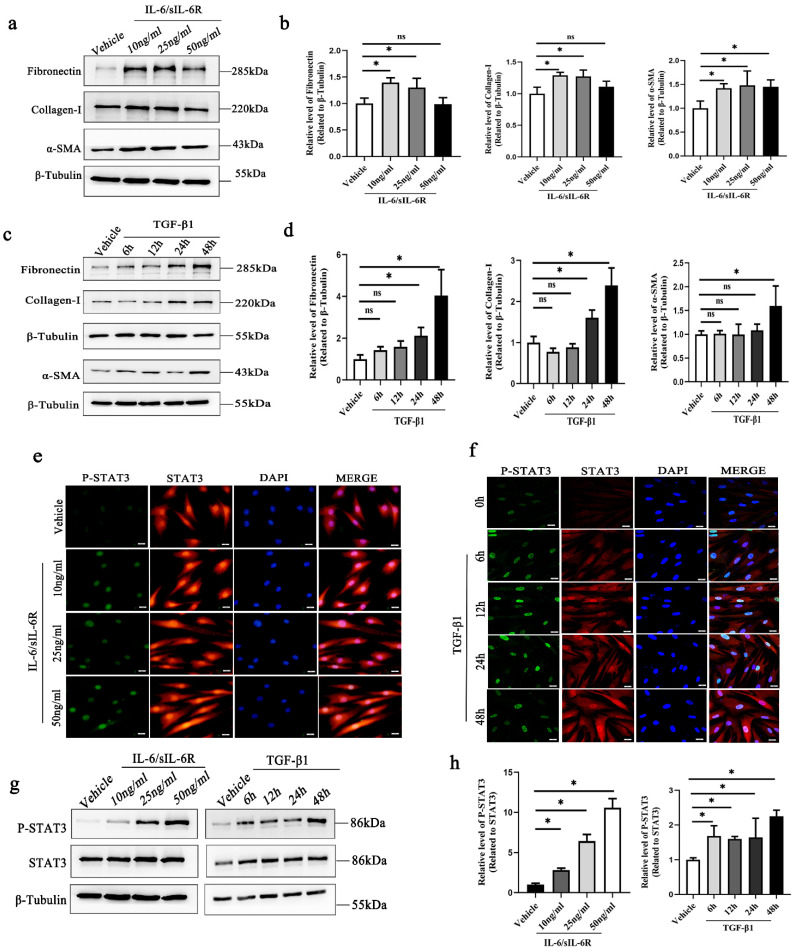
Increased expression of p-STAT3 in activated fibroblasts induced by IL-6 and TGF-β1. Western blot and quantitative analysis of the levels of fibronectin, collagen-I and α-SMA in fibroblasts after IL-6/sIL-6R (**a**,**b**) or TGF-β1 (**c**,**d**) stimulation. Immunofluorescence analysis (scale bar, 20 μm) of p-STAT3 (green) and total STAT3 (red) expression in cultured fibroblasts stimulated by IL-6/sIL-6R (**e**) or TGF-β1 (**f**). (**g**) Western blot and (**h**) quantitative analysis of p-STAT3 and total STAT3 in fibroblasts treated with TGF-β1 or IL-6/sIL-6R. * *p* < 0.05, ns *p* >0.05. Significance was determined by one-way ANOVA.

HTFs, which are key cells in subconjunctival fibrosis, are typically used to study fibrosis associated with GFS in vitro [20]. Activation of HTFs induced by various cytokines leads these cells to subsequently reenter the cell cycle, migrate and transform into myofibroblasts [21]. Myofibroblasts are characterized by the expression of α-SMA and produce collagen I and fibronectin, which are essential for ECM deposition [22]. Consistent with previous studies, our results demonstrated that the expression of collagen I, fibronectin and α-SMA was increased in response to IL-6/sIL-6R and TGF-β1 (Figure 1a–d). We further evaluated the level of phosphorylated STAT3 in the two complementary fibrosis models of HTFs: one involving inflammation-dependent pathways (induced by IL-6) and the other involving fibroblast activation devoid of inflammation (induced by TGF-β1). The results showed that the level of phosphorylated STAT3 was increased in IL-6- and TGF-β1-stimulated HTFs (Figure 1e–h). Immunofluorescence staining demonstrated nuclear localization of p-STAT3 and further confirmed the activation of STAT3 signaling in IL-6- and TGF-β1-stimulated fibroblasts (Figure 1e,f). Given the consistent activation of STAT3 in cultured HTFs induced by IL-6 and TGF-β1, we hypothesized that STAT3 was a core pathway for fibrogenesis induced by various cytokines.

### 2.2. STAT3 Regulates IL-6–and TGF-β1–Mediated Fibroblast Activation

**Figure 2:**
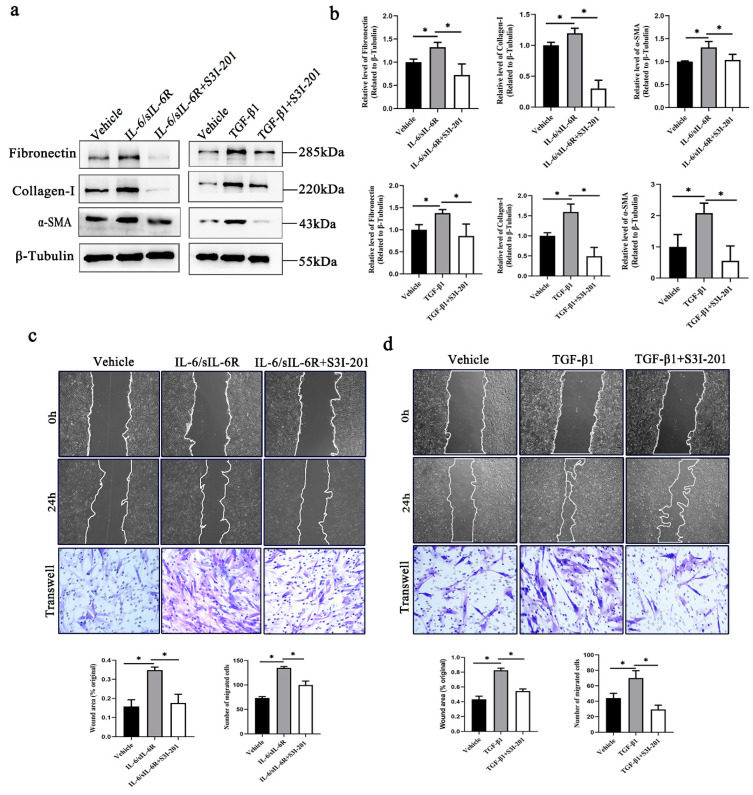
S3I-201 inhibits fibrogenesis in both IL-6/sIL-6R- and TGF-β1-cultured HTFs. (**a**) Western blot and (**b**) quantitative analysis of the expression of fibronectin, collagen-I and α-SMA in IL-6/sIL-6R- or TGF-β1-cultured fibroblasts treated with S3I-201. Wound healing (scale bar, 200 μm) and transwell chamber analysis (scale bar, 50 μm) of migration in (**c**) IL-6/sIL-6R- or (**d**) TGF-β1-cultured fibroblasts treated with S3I-201. * *p* < 0.05. Significance was determined by one-way ANOVA.

S3I-201, which is a small molecule inhibitor, can block STAT3 phosphorylation and STAT3 DNA binding by binding to the STAT3–SH2 domain. To further clarify the role of STAT3 in the fibrotic process induced by IL-6 and TGF-β1, we used S3I-201 to target STAT3 signaling in cultured HTFs treated with TGF-β1 or the IL-6/sIL-6R complex. Here, we found that collagen I, fibronectin and α-SMA levels in HTFs treated with S3I-201 were markedly reduced in both complementary fibrosis models. (Figure 2a,b). In addition, another well-known mechanism of subconjunctival fibrogenesis involves fibroblast migration from the periphery of the surgery site and proliferation in the resident location. In the present study, the chemotactic migration of fibroblasts was detected using wound healing assays and transwell chamber assays. We found that HTFs treated with S3I-201 exhibited less migration than those treated with IL-6 and TGF-β1 (Figure 2c,d). These data indicate that STAT3 signaling plays a central role in regulating IL-6–mediated and TGF-β1–mediated fibroblast activation, suggesting that STAT3 activation may integrate common profibrotic pathways to promote fibrosis.

### 2.3. SOCS3 Overexpression Suppresses STAT3 Activation and the Fibrotic Response Mediated by TGF-β1 and IL-6

**Figure 3:**
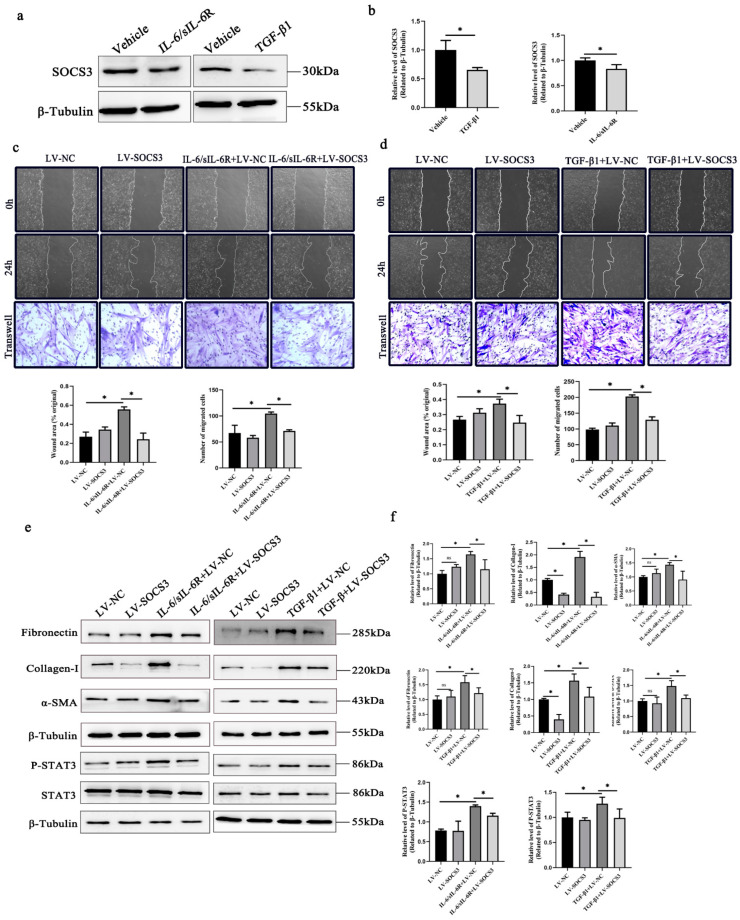
SOCS3 overexpression inhibits STAT3 activation and fibrogenesis in IL-6/sIL-6R- and TGF-β1-cultured HTFs. (**a**) Western blot and (**b**) quantitative analysis of the expression of SOCS3 in IL-6/sIL-6R- or TGF-β1-cultured fibroblasts. Wound healing (scale bar, 200 μm) and transwell chamber analysis (scale bar, 50 μm) of migration in (**c**) IL-6/sIL-6R- or (**d**) TGF-β1-cultured overexpressed SOCS3 fibroblasts. (**e**) Western blot and (**f**) quantitative analysis of the expression of fibronectin, collagen-I, α-SMA, P-STAT3 and total STAT3 in IL-6/sIL-6R- or TGF-β1-cultured overexpressed SOCS3 fibroblasts. * *p* < 0.05, ns *p* >0.05. Significance was determined by one-way ANOVA.

Previous studies have shown that STAT3 plays a key role in the fibrotic response of HTFs. Suppressors of cytokine signaling 3 (SOCS3) can bind to JAKs to inhibit their kinase activity, preventing subsequent activation of STAT3 and the transcription of STAT3-dependent target genes [23]. We tested the hypothesis that SOCS3 may negatively regulate fibroblast activation by inhibiting STAT3 signaling induced by TGF-β1 or IL-6. The results showed that the expression of SOCS3 was downregulated in cultured HTFs treated with TGF-β1 and the IL-6/sIL-6R complex (Figure 3a,b). To further confirm the role of SOCS3 in the fibrotic process, SOCS3 was overexpressed in HTFs through lentivirus infection. We found that the expression of p-STAT3 was decreased, and the activated phenotype of fibroblasts was completely rescued. More specifically, SOCS3 overexpression significantly reversed the protein expression of collagen, fibronectin and α-SMA in HTFs treated with TGF-β1 or the IL-6/sIL-6R complex (Figure 3e,f). Furthermore, the effect of TGF-β1 or IL-6 on the migration of HTFs cells was prevented by SOCS3 overexpression (Figure 3c,d).

### 2.4. S3I-201 Is Safe for Subconjunctival Injection in Rat Eyes

**Figure 4:**
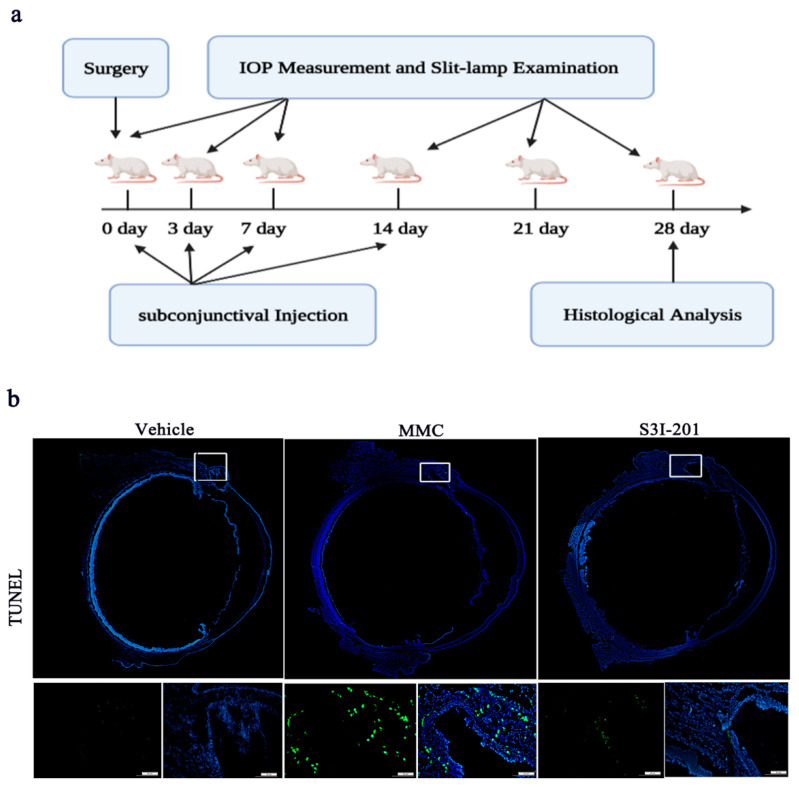
Assessment of the toxicity of S3I-201 in rat eyes. (**a**) Experimental scheme for the rats after GFS. (**b**) Representative images of TUNEL staining (upper panel scale bar, 200 μm; lower panel scale bar, 20 μm) of the subconjunctival tissues of surrounding the filtering areas.

MMC, which is a cytotoxic antiproliferative agent, is typically used to reduce scarring following GFS [24]. However, the failure rate is still approximately 50% at 5 years [25]. Moreover, the potential risk of complications such as endophthalmitis and corneoscleral toxic effects limits the long-term use of these agents [26]. We sought to determine the potential safety of S3I-201 in treating subconjunctival scar formation in a rat model of GFS. Rats with GFS were randomly assigned to three groups, and each group of animals was treated with S3I-201, MMC, or DMSO (designated the vehicle group). The treatment scheme is shown in Figure 4a. A TUNEL assay was used to evaluate the toxicity of S3I-201 and MMC to rat eyes after GFS. As expected, many TUNEL-positive cells in the subconjunctival area were observed in the MMC group. In contrast, few conjunctival TUNEL-positive cells were found in the S3I-201 group (Figure 4b). Based on this observation, we concluded that S3I-201 had almost no toxic effects on the normal ocular tissues of rats.

### 2.5. S3I-201 Treatment Ameliorates Rebound IOP Elevation and Prolongs Filtering Bleb Survival after GFS

**Figure 5:**
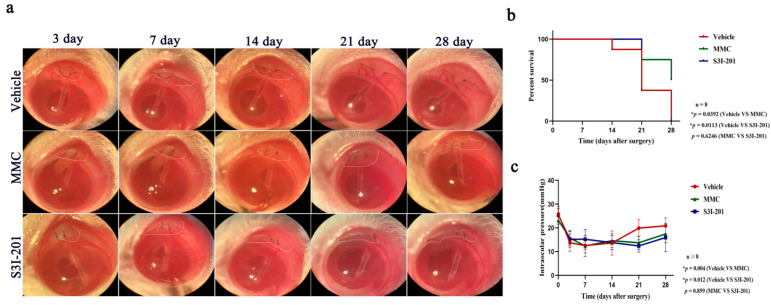
S3I-201 promoted functional bleb formation and maintained the decrease in IOP for a period in rats after GFS. (**a**) Representative morphological images of filtering blebs at different time points in the vehicle, MMC and SI-201 treatment groups; bleb area (white line). (**b**) Kaplan–Meier survival curve of filtering blebs in the vehicle, MMC and SI-201 treatment groups (log rank test; overall *p* < 0.05, vehicle vs. MMC *p* < 0.05, vehicle vs. S3I-201 *p* < 0.05, * *p* < 0.05). (**c**) IOPs of the eyes at different time points in the vehicle, MMC and SI-201 treatment groups (repeated measure ANOVA; vehicle vs. MMC *p* < 0.05, vehicle vs. S3I-201 *p* < 0.05, * *p* < 0.05).

The obstruction of aqueous humor outflow resulting from subconjunctival fibrosis following GFS leads to rebound IOP elevation, which is the main factor responsible for the failure of filtering surgery. To ascertain the potential effect of S3I-201 treatment on subconjunctival scar formation, we first evaluated the effect of S3I-201 treatment on the stabilization of the reduction in IOP in a rat model of GFS. After GFS, a drastic decrease in IOP was observed on postoperative day 3 in animals in all three surgical groups (vehicle 13.7 ± 1.1 mmHg, MMC 14.2 ± 1.2 mmHg, S3I-201 15.7 ± 1.1 mmHg). Animals in the vehicle group showed a complete rebound IOP elevation to a level equivalent to the baseline on days 21 to 28. Animals in the S3I-201- and MMC-treated groups also exhibited a rebound IOP elevation. However, the rebound occurred at a slower rate than that in the vehicle group. S3I-201-treated rats showed the slowest rate of rebound IOP elevation during the 28 days after GFS (Figure 5c).

Maintenance of a functional filtering bleb is crucial for IOP control following GFS. Thus, we evaluated the effect of S3I-201 treatment on the survival of filtering blebs following GFS. Bleb survival analysis demonstrated a significant difference in the survival distribution among the vehicle, MMC, and S3I-201 groups (*p* < 0.05). Rats in the vehicle group exhibited rapid loss of filtering blebs after GFS, and the filtering blebs were completely lost in this group on day 28. Compared with the vehicle group, filtering blebs in the MMC and S3I-201 groups survived a significantly longer period of time. Loss of filtering blebs in the MMC group started on day 14, and the survival rate of filtering blebs was 50% on day 28. The loss of blebs in the S3I-201 group occurred on day 21, and the survival rate of filtering blebs was 62.5% on day 28. S3I-201 and MMC treatment significantly improved the survival of blebs compared with the vehicle group (Figure 5a,b). Collectively, these data suggest that S3I-201 treatment benefits the stabilization of IOP reduction and the survival of filtering blebs after GFS.

### 2.6. S3I-201 Treatment Alleviates Subconjunctival Fibrosis following GFS

**Figure 6:**
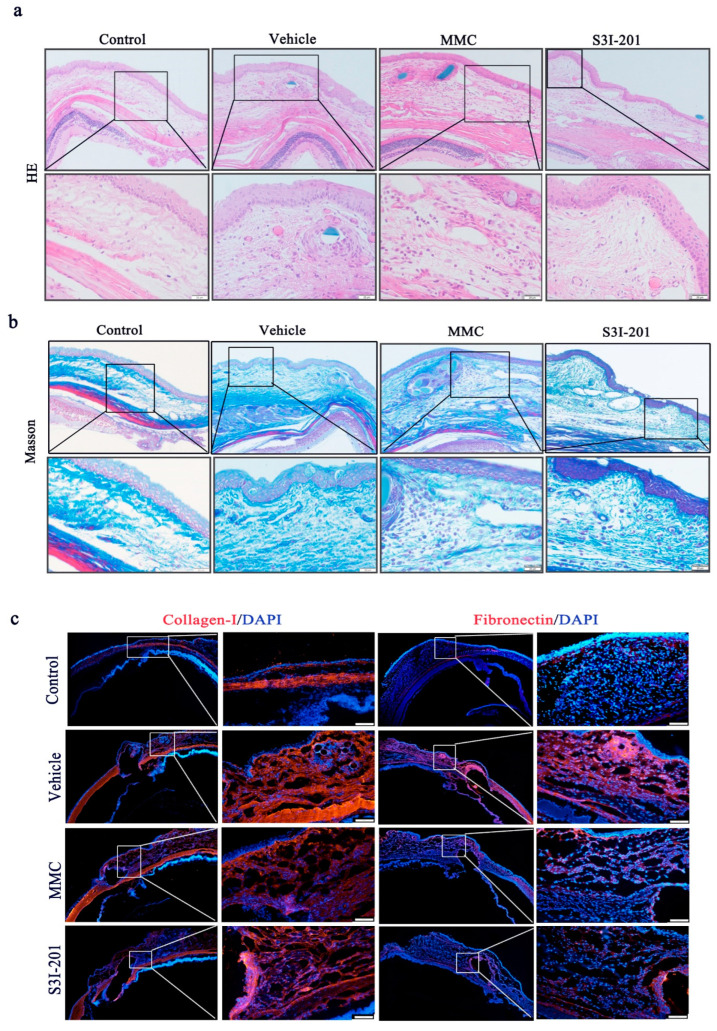
S3I-201 attenuated subconjunctival fibrosis in rats after GFS. (**a**) Representative HE staining images (upper panel scale bar, 100 μm; lower panel scale bar, 20 μm) and (**b**) Masson’s trichrome staining images (upper panel scale bar, 100 μm; lower panel scale bar, 20 μm) of the operative area. (**c**) Representative immunofluorescent images (left panel scale bar, 200 μm; right panel scale bar, 20 μm) for specific markers, including collagen-I, fibronectin (red) and DAPI (blue), in the subconjunctival tissues of surrounding filtering areas.

To observe the pathological changes in the subconjunctival tissues following GFS, HE and Masson staining were performed. HE staining showed that the subconjunctival tissue in the vehicle group was significantly thickened with marked hyperplasia of fibrous connective tissue, inflammatory cells and fibroblasts were of a high density and grew in clumps. Compared with the control group (no surgery), Masson staining showed that collagen deposition was increased in the vehicle group. In the MMC group, the subconjunctival fibrous layer was looser and thinner, forming cavities, with few cells and reduced collagen deposition. In the S3I-201 group, the structure of fibrous connective tissue was loose, with the formation of voids, few cells and reduced collagen deposition, as observed by HE and Masson staining (Figure 6a,b).

To further investigate the subconjunctival fibrotic response to surgical injury induced by GFS, immunofluorescence was used to analyze the expression of collagen-I and fibronectin in the bleb tissues of rat eyes. Immunofluorescence analysis showed that the expression of collagen-I and fibronectin was significantly increased after GFS in the vehicle group, indicating that GFS activated the expression of profibrotic genes. MMC and S3I-201 treatment significantly attenuated surgical injury-induced expression of these genes, suggesting that similar to MMC, S3I-201 inhibits subconjunctival scar formation after GFS. Consistent with the Masson staining results, immunofluorescence staining demonstrated less collagen deposition in the subconjunctival region after GFS in the S3I-201 and MMC groups than in the vehicle group (Figure 6c).

## 3. Discussion

In the current study, we identified STAT3 as a key intracellular mediator of profibrotic effects. Inhibiting STAT3 in cultured HTFs by pharmacological inactivation or siRNA prevents fibroblast proliferation and migration and the differentiation of resting fibroblasts into myofibroblasts and significantly reduces the deposition of ECM. It was also demonstrated that STAT3 signaling plays a central role in regulating IL-6–and TGF-β–mediated fibroblast activation, suggesting that STAT3 activation may integrate common profibrotic pathways to promote fibrosis. Finally, we provided in vivo evidence that pharmacological inactivation of STAT3 inhibits profibrotic gene expression and subconjunctival fibrosis in a rat model of GFS. Together, these data demonstrate that STAT3 is a core pathway for fibrogenesis.

The current study used in vitro fibrosis models induced by IL-6 and TGF-β1 to advance our understanding of how STAT3 may regulate the development of fibrosis. These models allow for the investigation of different phases or pathways involved in wound healing. The acute wound healing process after trauma or surgery, including GFS, is traditionally divided into four overlapping phases: hemostasis, inflammation, proliferation, and remodeling [27]. In the inflammatory phase, recruited neutrophils and macrophages secrete proinflammatory cytokines, such as IL-6, into wound sites [28,29]. Subsequently, the proliferative phase of wound healing is initiated by an influx of fibroblasts and is characterized by the transdifferentiation of fibroblasts into myofibroblasts, which is driven by TGF-β [30]. In vitro studies with HTFs clearly demonstrate that STAT3 activation can regulate the fibrotic phenotype of HTFs triggered by IL-6 or TGF-β1. These data suggested that inhibiting STAT3 decreases fibrosis in the inflammatory phase and proliferative phase during the wound healing process.

STAT3 signaling is strictly regulated by the suppressor of cytokine signaling 3 (SOCS3) proteins [31]. Hence, we hypothesized that fibroblast activation induced by TGF-β1 or IL-6 may be associated with the suppression of SOCS3. Our results showed that the expression of SOCS3 was downregulated in HTFs treated with IL-6 and TGF-β1. To further confirm that SOCS3 participates in fibrosis mediated by STAT3 activation, SOCS3 was overexpressed in HTFs. Indeed, we found that SOCS3 overexpression significantly rescued the fibrotic responses induced by IL-6 and TGF-β1 due to the inactivation of STAT3. These data suggested that SOCS3 may play a suppressive role in fibrosis promoted by STAT3.

S3I-201 is a selective and potent inhibitor that binds to the SH2 domain of STAT3 to block the constitutive activation of STAT3 and the expression of STAT3 target genes [32]. S3I-201 inhibits transcriptional activity in cells that contain constitutive STAT3 activation by blocking STAT3–STAT3 complex formation and STAT3 DNA binding. However, other commonly used STAT3 inhibitors, such as AG490, act on Janus kinases, which are upstream activators of STAT3, and other STAT isoforms [33,34]. Unlike STAT3, recent studies using knockout mice showed that STAT1 and STAT5 play key roles in promoting liver and lung fibrosis after injury [35,36]. Thus, S3I-201 was used to treat GFS fibrosis in our study because its characteristics distinguished it from other STAT3 inhibitors.

Fibrosis in filtering area prevention is critical for the success of GFS. In this study, S3I-201 significantly reduced the IOP and prolonged bleb survival compared with the vehicle. Histologic examination showed that the numbers of inflammatory cells and myofibroblasts were reduced in the S3I-201 group compared with the vehicle group. Collagen deposition was also reduced in the S3I-201 group. Additionally, only a few TUNEL-positive cells were observed in the conjunctiva of the S3I-201-treated group; conversely, many TUNEL-positive cells were observed in the MMC group due to significant toxicity. These results indicate that S3I-201 was safe as an antiproliferative medication for use in the GFS rat model. The in vivo and in vitro results strongly suggest that STAT3 is a potential target in fibrotic responses after GFS.

However, importantly, this study has its own limitations. First, the optimal dosage, concentration, and application method of S3I-201 are needed to further improve the surgical outcome after GFS. Second, the sample size was small, and the follow-up time was short. More samples and longer follow-ups are needed in future studies. Finally, the inhibitory effect of S3I-201 on GFS was not superior to MMC. However, S3I-201 was observed to have a less toxic effect on eye tissues than MMC. We can expect that the use of S3I-201 can improve the safety profile of surgery compared with MMC. Additionally, S3I-201 combined with MMC may further inhibit the fibrotic response after GFS and lower the dose and exposure time of MMC.

In conclusion, STAT3 integrates profibrotic signals from different mechanisms, suggesting that STAT3 may be a promising target for antifibrotic therapies following GFS. Indeed, the findings of the current study demonstrate that inactivating STAT3 signaling exerts potent antifibrotic effects on GFS-induced fibrosis in vitro and in vivo. Furthermore, STAT3 inactivation inhibits IL-6-induced fibrosis in the early inflammatory phases of wound healing but also exerts potent antifibrotic effects on TGF-β-induced fibrosis in the late noninflammatory stages of wound healing. Thus, targeting STAT3 may be beneficial for different wound healing stages in subconjunctival fibrosis following GFS.

## 4. Materials and Methods

### 4.1. The Preparation of Tissues and Cells

The human subconjunctival Tenon’s capsules that were used to isolate HTFs were obtained from patients undergoing strabotomy. Under sterile conditions, the tissues were cut into 3 to 4 mm pieces. The samples were then placed into a fibroblast medium (FM) (ScienCell, San Diego, CA, USA) containing 10% fetal bovine serum (FBS), 100 U/mL penicillin, 100 µg/mL streptomycin and fibroblast growth supplement. Once the growth of the HTFs was well established, the cells were passaged in a monolayer after being treated with trypsin/EDTA. Cells that were between the third and fifth passages were used in the studies. The study followed the principles outlined in the Helsinki Declaration and was examined and approved by the Second Hospital of Jilin University’s Institutional Review Board.

### 4.2. Cell Stimulation

HTFs were stimulated with recombinant TGF-β1 (R&D Systems, Minneapolis, MN, USA), recombinant IL-6 (R&D Systems, Minneapolis, MN, USA) and recombinant sIL-6R (R&D Systems, Minneapolis, MN, USA). sIL-6R is required for intracellular IL-6 signal transduction because mIL-6R is not expressed by HTFs [37].

### 4.3. Overexpression Experiment

GeneCopoeia (Guangzhou, China) provided lentiviruses to overexpress SOCS3 or those containing a control vector. Lentivirus-containing media was added and mixed with the HTFs once the cells reached 30% confluence in 6-well plates. Supernatants in the wells were removed after 16 h of incubation and replaced with fresh FM, and the cells were then cultured for 48 h for further analysis.

### 4.4. Western Blot

Protein samples were separated by SDS-polyacrylamide gel electrophoresis and electrotransferred onto polyvinylidene fluoride (PVDF) membranes (Merck Millipore, Burlington, MA, USA). After being blocked, the membranes were incubated with rabbit anti-collagen I (clone E8F4L, catalog 72026, Cell Signaling Technology, Danvers, MA, USA), rabbit anti-fibronectin (clone EPR23110-46, catalog ab268020, Abcam, Cambridge, UK), rabbit anti-α-SMA (clone EPR5368, catalog ab124964, Abcam, Cambridge, UK), rabbit anti-SOCS3 (polyclonal, catalog AF8025, Beyotime, Shanghai, China), mouse anti-STAT3 (clone 124H6, catalog 9139, Cell Signaling Technology, Danvers, MA, USA), or rabbit anti–p-STAT3 (Tyr705) (clone D3A7, catalog 9145, Cell Signaling Technology, Danvers, MA, USA) overnight at 4 °C. Equal protein loading was confirmed by incubation with mouse anti–β-tubulin (clone 5G3, catalog 44032, Signalway antibody, Greenbelt, MD, USA). Horseradish peroxidase-conjugated secondary antibodies (Signalway antibody, Greenbelt, MD, USA) were incubated with the membranes at room temperature for 1 h. Finally, the bands were visualized with electrochemiluminescence (Merck Millipore, Burlington, MA, USA) and analyzed by ImageJ software 1.46r.

### 4.5. Immunofluorescence Staining

Tissue sections or cultured HTFs were stained with rabbit anti-collagen I (clone E8F4L, catalog 72026, Cell Signaling Technology, Greenbelt, MD, USA), rabbit anti-fibronectin (clone EPR23110-46, catalog ab268020, Abcam, Cambridge, UK), mouse anti-STAT3 (clone 124H6, catalog 9139, Cell Signaling Technology, Greenbelt, MD, USA), rabbit anti–p-STAT3 (Tyr705) (clone D3A7, catalog 9145, Cell Signaling Technology, Greenbelt, MD, USA), and DAPI (catalog C0060, Solarbio, Beijing, China). The samples were analyzed using a fluorescence microscope (Olympus Corporation, Tokyo, Japan).

### 4.6. Wound Healing Assay

First, the supernatant of HTFs was aspirated, and a wound was created with a sterile 200 µL pipette tip in the bottom of the 6-well plates. Subsequently, the cells were washed twice with PBS and immediately photographed with an inverted microscope (Olympus Corporation, Tokyo, Japan). Following 24 h of incubation, images were taken again, and the migration distance was calculated. The formula for calculating the cell migration rates is as follows: cell migration rate (%) = (the distance prior to healing − the distance following healing/the distance prior to healing) × 100%.

### 4.7. Transwell Chamber Assay

Trypsin was used to digest the HTFs in each group, and 600 µL of medium containing 20% FBS was added to the lower chamber of a transwell chamber (Labselect, Hangzhou, China) in a 24-well plate. Next, the cells were resuspended in a serum-free medium and transferred to the upper chamber (1 × 10^4^ cells/well). After 24 h of incubation at 37 °C, the cells that had not invaded from the upper chamber were removed with a cotton swab. Paraformaldehyde and crystal violet staining solutions were used to fix and stain the invasive cells in the lower chamber at room temperature for 20 min. Five fields were randomly selected and photographed with an inverted microscope. The number of invading cells was counted by ImageJ software 1.46r.

### 4.8. Animal Experiments

Adult male Sprague–Dawley rats that weighed 180–200 g were purchased from Changchun Yisi Biotechnology. The rats were anesthetized by an intraperitoneal injection of 3 mL/kg 10% chloral hydrate followed by ocular surface anesthesia using 0.5% oxybuprocaine hydrochloride eye drops (Santen, Osaka, Japan). All animals were treated in accordance with the ARVO Statement for the Use of Animals in Ophthalmic and Vision Research.

GFS was performed on bilateral eyes as previously described [38,39]. To create a conjunctival fornix-based conjunctival flap 3–5 mm behind the limbus, a conjunctival incision was made, and then the underlying Tenon’s capsule was bluntly dissected. Subsequently, a 30-G needle was inserted into the anterior chamber to create a full-thickness scleral tunnel. During the process, great care was taken to avoid iridal blood vessels. Viscoelastic solution (Alcon, Fort Worth, TX, USA) was injected through the needle to maintain the depth of the anterior chamber. A beveled micro cannula (external diameter, 0.3 mm) was then inserted through the scleral tunnel. Finally, the conjunctiva and Tenon’s capsule were closed using a 10-0 monofilament nylon suture (Ethicon, Suzhou, China). All surgeries were performed by the same surgeon. Eyes exhibiting subsequent slippage or dislocation of the cannula were excluded.

After surgery, the eyes were treated with dimethyl sulfoxide (DMSO) as a vehicle, S3I-201 (Sigma–Aldrich, Burlington, MA, USA), and MMC (Hanhui, Shanghai, China) (n ≥ 8) separately. S3I-201 (10 mg/mL, 5 μL) was injected into the subconjunctival space with a 10-μL Hamilton syringe connected to a 33-G needle (Hamilton Company, Reno, NV, USA) immediately after surgery and on days 3, 7, and 14. MMC was applied at a dose of 0.4 mg/mL with a small piece of cellulose sponge for 5 min. Then, irrigation of the treated area was performed with 2 mL 0.9% sodium chloride using a syringe. Each treatment was performed on at least 8 eyes (Vehicle group n = 10; S3I-201 group n = 11; MMC group n = 8). The IOP, bleb appearance and survival, and complications were observed on days 3, 7 14, 21 and 28. At the end of the experiment, 8 eyes were collected for histochemical and immunofluorescence analyses.

### 4.9. Clinical Examination and Analysis of Blebs

The rats were anesthetized prior to IOP measurement. Intraocular pressure was measured in each rat with a Tono-Pen tonometer (Reichert, Depew, NY, USA) according to the manufacturer’s instructions. All IOP measurements were taken 5 to 10 times in each eye.

Slit-lamp examinations were performed on subconjunctival bleb morphology and determined survival time. A bleb was judged to have failed if the surgical site appeared flat and vascularized by slit-lamp analysis.

### 4.10. Hematoxylin and Eosin (HE) and Masson’s Trichrome Staining

On day 28 after surgery, the rats were euthanized, and both eyes were immediately enucleated. The eyes were fixed in FAS fixative (Wuhan Servicebio, Wuhan, China), which contains formaldehyde, acetic acid, and saline, for 48 h, and then embedded in paraffin. The eyes were cut into sequential 4 μm tissue sections and then dewaxed with xylene. The sections were analyzed by HE and Masson’s trichrome staining to evaluate histopathology and collagen expression in the subconjunctival tissues surrounding the filtration passage. Representative images were taken using an inverted microscope.

### 4.11. TUNEL Assay

Tissue sections of rat eyes were processed with a TUNEL kit (Beyotime, Shanghai, China) according to the manufacturer’s instructions, and the images were analyzed using fluorescence microscopy.

### 4.12. Statistical Analysis

The results are presented as the means ± standard deviations (SD) of at least three independent experiments. One-way analysis of variance (ANOVA) and *t*-tests were performed with SPSS to analyze the statistical results. Bleb survival analysis was performed with Kaplan–Meier and Mantel–Cox pairwise comparison tests. Values of *p* < 0.05 were considered significant.

## Data Availability

The novel findings reported in this study can be found within the article. For additional information or inquiries, please contact the corresponding author.
